# Constitutive Modeling of the Densification Behavior in Open-Porous Cellular Solids

**DOI:** 10.3390/ma14112731

**Published:** 2021-05-21

**Authors:** Ameya Rege

**Affiliations:** German Aerospace Center (DLR), Department of Aerogels and Aerogel Composites, Institute of Materials Research, Linder Höhe, 51147 Cologne, Germany; ameya.rege@dlr.de

**Keywords:** constitutive model, cellular material, cell wall mechanics, pore-collapse, densification

## Abstract

The macroscopic mechanical behavior of open-porous cellular materials is dictated by the geometric and material properties of their microscopic cell walls. The overall compressive response of such materials is divided into three regimes, namely, the linear elastic, plateau and densification. In this paper, a constitutive model is presented, which captures not only the linear elastic regime and the subsequent pore-collapse, but is also shown to be capable of capturing the hardening upon the densification of the network. Here, the network is considered to be made up of idealized square-shaped cells, whose cell walls undergo bending and buckling under compression. Depending on the choice of damage criterion, viz. elastic buckling or irreversible bending, the cell walls collapse. These collapsed cells are then assumed to behave as nonlinear springs, acting as a foundation to the elastic network of active open cells. To this end, the network is decomposed into an active network and a collapsed one. The compressive strain at the onset of densification is then shown to be quantified by the point of intersection of the two network stress-strain curves. A parameter sensitivity analysis is presented to demonstrate the range of different material characteristics that the model is capable of capturing. The proposed constitutive model is further validated against two different types of nanoporous materials and shows good agreement.

## 1. Introduction

Open-porous cellular materials exhibit remarkable mechanical properties for their light-weight structural features. While many materials found in the nature, e.g., wood, bones, cork, coral, exhibit cellular characteristics, many man-made materials have been designed specifically with such characteristics, namely, polymeric and glass foams, aerogels, honeycombs. Most of the man-made foams have been developed for thermal insulation applications, owing to their low thermal mass which is proportional to their relative density. However, many recent applications of such materials have targeted structural functions, such as sandwich panels in light-weight design. Cellular materials maximize their mechanical properties with increasing densities. This is true for properties such as the Young’s modulus or the yield stress. The relation between the Young’s modulus *E* and the density ρ can be expressed as [1],

(1) $$\frac{E_{\mathrm{bulk}}}{E_{\mathrm{skel}}}\propto {\left(\frac{\rho_{\mathrm{bulk}}}{\rho_{\mathrm{skel}}}\right)}^{m},$$

where ${•}_{\mathrm{bulk}}$ and ${•}_{\mathrm{skel}}$ denote the bulk and skeletal properties and *m* represents the scaling exponent. The structure-property relations in porous materials have been well investigated by Gibson and Ashby [1], and form a basis for several other works. According to their open-cell foam model, m=2 for a perfectly connected porous solid. However, many materials such as aerogels and porous carbon materials exhibit 1<m<4 [2,3].

With regards to modeling the mechanical behavior of open-porous materials, there exist diverse approaches, namely, description based on their cell wall properties by using simplified beam theory-based models or description based on the mean field homogenization schemes, typically used for materials with voids. In the first approach, the macroscopic mechanical properties of such materials are modeled based on the geometric and material properties of their microscopic cell walls. This formed the basis for deriving Equation (1). These cell walls are typically idealized as quadratic or cylindrical beams. Focussing on the classical beam theory-based models, the idealization of the cell shapes is generally based on simple two-dimensional (2-d) ones (e.g., triangle, square, hexagonal cells) or more complex three-dimensional (3-d) ones (e.g., cubic, rhombic dodecahedron, tetrakaidecahedron cells). Gibson et al. [4] presented a 2-d analysis of foam-like open-porous cellular materials, by describing their bulk mechanical properties as functions of the microscopic cell wall properties, where the cells were assumed to be of a hexagonal shape, mimicking honeycombs. Elementary Euler-Bernoulli beam theory was used to present the mathematical expressions. Parameters such as the Young’s modulus of the bulk material, Poisson ratio and shear deflection were mathematically derived. Davini and Ongaro [5] further developed a homogenized model, generalizng the results obtained in [4], for describing the mechanical behavior of 2-d cellular foams from honeycombs. In another report, Gibson and Ashby [6] presented a similar analysis for 3-d cellular materials. There, they presented a description of the linear and nonlinear elastic and plastic behavior of such materials by means of the Euler-Bernoulli beam theory. In both, the 2-d and 3-d cases, good agreement with the experimentally obtained properties was found. Several studies have built up on these theories for developing models for more specific cases. For example, Zhu et al. [7] modeled open-cell foams using more complex tetrakaidecahedral cells.

All of the above-mentioned studies describe the bulk mechanical properties in terms of the geometric and mechanical properties of their struts. However, very few papers report on modeling the mechanical stress-strain behavior of the materials. A micromechanical modeling approach to describe the compressive behavior of nanoporous aerogel-based materials was described in Rege et al. [8]. There, the aerogel network was assumed to be made up of idealized square-shaped cells, those that are homogeneously distributed through the network. The generalization to 3-d was achieved by directional averaging. The mechanical behavior of the cell walls was modeled using the Euler-Bernoulli beam theory for large deflections [9]. A numerical approach to describe large deformations was used [10], and the model showed good agreement with the experimental data. In another study [11], the model was improved and an analytical solution for large deflections based on elliptic integrals was derived and the model showed good agreement not only with aerogels from cellulose, but also from pectin and k-carrageenan. These models were shown to describe the linear elastic regime and the subsequent pore collapse. The pores were considered to collapse once the bending stress in the cell walls reached a predefined critical value. Rege and Itskov [12] then proposed an approach to model the tensile behavior of such materials. The model was able to capture not only the nature of the stress-strain curve but could also predict the network failure with high accuracy. A generalized model to describe the mechanical behavior of open-porous cellular materials with elastic, ductile and brittle properties was very recently published in Rege et al. [13], where the influence of the pore-size distributions in nanoporous materials, together with the mode of deformation in the pore walls, on the bulk mechanical properties was presented. However, while describing the compressive behavior, the model only captured the linear elastic regime and the subsequent pore collapse. A hardening mechanism, that results from the densification of the network, was found to be missing in these previous reports.

Gibson and Ashby [1] proposed that one could equate the limiting strain (strain at which the onset of densification appears, $\epsilon_{D}$) to the material’s porosity, because this is the point at which all the pore space has been squeezed out. However, they showed that the cell walls lock together much sooner and proposed an empirical relation,

(2) $$\epsilon_{D}=1-1.4\frac{\rho_{\mathrm{bulk}}}{\rho_{\mathrm{skel}}},$$

which has been found to be not consistent for every porous material. Thus, the parameter $\epsilon_{D}$ has also been investigated by several other authors. Li et al. [14] investigated different approaches used to interpret $\epsilon_{D}$ and found that the method based on the energy absorption efficiency curve gave most consistent results.

In all the above-mentioned studies, the shape of the cells was assumed to be constant through the structure. While the models proposed by Rege et al. [8,11,12,13] account for the variation in cell sizes, they also assumed an idealized cell shape. On the one hand Kraynik et al. [15] established that the elastic response of such materials was insensitive to the variability in the cell sizes and shapes, while on the other hand, Rege et al. [13] showed that the pore sizes play a significant role in dictating the mechanical properties of nanoporous cellular materials. To find a solution towards modeling such materials by accounting for the variability in the cell structure, an alternative approach is presented in the form of finite element methods [16,17,18]. There, Voronoi-based modeling approaches are found widely in the literature [19,20,21,22]. By subjecting the Voronoi-based representative volume elements to large deformations, the densification behavior can be captured. This then not only predicts $\epsilon_{D}$, but can also capture the stress-strain curve under densification. However, in this paper, the focus is on numerical approaches and a reader interested in Voronoi-based methods, may refer to the references cited above. In this paper, the micromechanical model approach presented in [13] has been extended to capture the hardening in the compressive stress-strain curve during the densification of the material network.

The paper is organized as follows. In Section 2, the constitutive model is proposed. The results from the model are presented and discussed in Section 3. There, the model is also validated with a few nanoporous materials. Finally, a conclusion is presented in Section 4.

## 2. Constitutive Model

**Figure 1:**
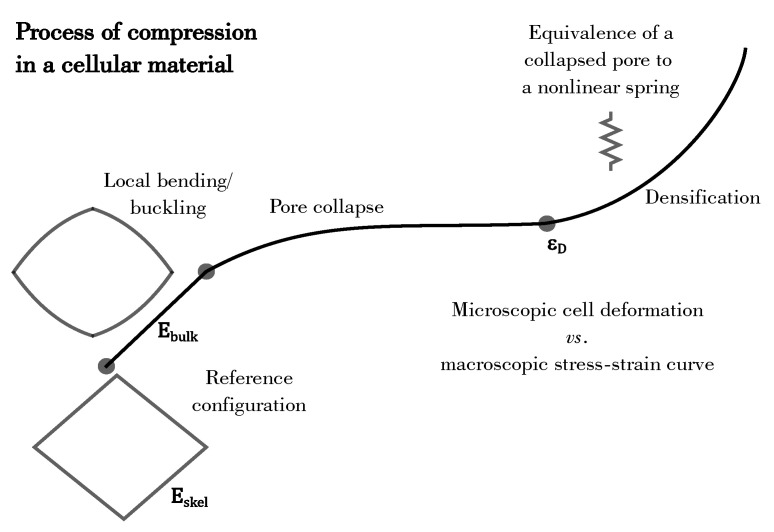
Scheme of the process of compression of a cellular solid. The black curve shows the stress-strain response, exhibiting the linear elastic regime, the plateau regime and the densification regime. Correspondingly, the deformation in a cell is illustratively shown.

Figure 1 illustrates the typical stress-strain curve in an open-porous cellular material. The entire deformation process can be divided into three regimes: (1) linear elastic regime, (2) plateau regime, and (3) densification regime. In the first regime, local bending and buckling occurs in the pore walls, followed by a pore collapse in the second regime. Lastly, the collapsed pores begin to densify resulting in hardening of the network. Accordingly, the proposed constitutive model is based on the following assumptions: (a) the open-porous network is made up of idealized square shaped cells that are homogeneously distributed through the complete network and (b) the collapsed cells act as a foundation to the undamaged cells. The model is described as follows.

### 2.1. Strain Energy Functions

Considering the cell wall as an Euler-Bernoulli beam, the bending and stretching strain energy functions can be fundamentally expressed as [13]

(3) $$\begin{array}{cc} \psi_{b} & =\int_{0}^{l}\frac{1}{2}\kappa {\left(\phi^{\prime }\right)}^{2}dl, \end{array}$$

(4) $$\begin{array}{cc} \psi_{s} & =\int_{0}^{l}\frac{1}{2}\mu {\left(u^{\prime }\right)}^{2}dl, \end{array}$$

where $\phi^{\prime }$ is the curvature and $u^{\prime }$ is the axial strain along the cell walls. Also, κ and μ represent the bending and stretching stiffness in the cell walls, respectively. Since the square-shaped cell is fully symmetric, the strain energy in a cell ($\psi_{o}$) is then four times the strain energy in a cell wall, which is a sum of the corresponding bending and stretching energies [8],

(5) $$\psi_{o}=4\cdot \left(\psi_{b}+\psi_{s}\right).$$

The damage in the pore-walls is based on the criteria outlined in Rege et al. [13]. This means that depending on the type of material being modeled, viz., elastic, ductile or brittle, the choice of the damage criterion can be made. Since the goal of this study was not to present the previously investigated effect of the different damage criteria, the results presented in this study have used the irreversible bending mode of deformation. This means that the cell walls were considered to undergo irreversible deformation after the bending moment of the pore-wall reached its maximal allowable value. Beyond this point, the yield stress in the pore-wall is reached, and a plastic hinge would likely appear. It is very difficult to micromechanically describe the deformation of a collapsed pore as it involves highly nonlinear deformations in the walls, ending up with a very complex geometrical structure. However, it seems feasible to consider that these collapsed cells form a foundation to the active cells undergoing deformation. Then, to simplify the problem, we assume the collapsed cell to act as a nonlinear spring. This is because the collapsed cell resists the compressive deformation, which subsequently results in the hardening of the network. The concept of modeling certain effects in cellular materials using springs is not unknown, as Ongaro et al. [23] modeled the behavior of filled cellular materials by using springs as foundations to the cell walls to provide resistivity to compression. In this work, we instead model a collapsed cell as a nonlinear spring. The correlation length of the spring ζ is considered to be equal to *l*. The force in one such collapsed cell can be expressed as

(6) $$F_{\zeta }=\frac{1}{\zeta }\frac{\partial \psi_{\zeta }}{\partial \lambda_{\zeta }},$$

where $\lambda_{\zeta }$ denotes the micro-stretch in the spring. The strain energy ($\psi_{c}$) of such a collapsed cell is expressed as

(7) $$\psi_{c}=\int_{\lambda_{\zeta }}k\cdot \zeta^{2}\left(1-\lambda_{\zeta }\right)d\lambda_{\zeta },$$

**Figure 2:**
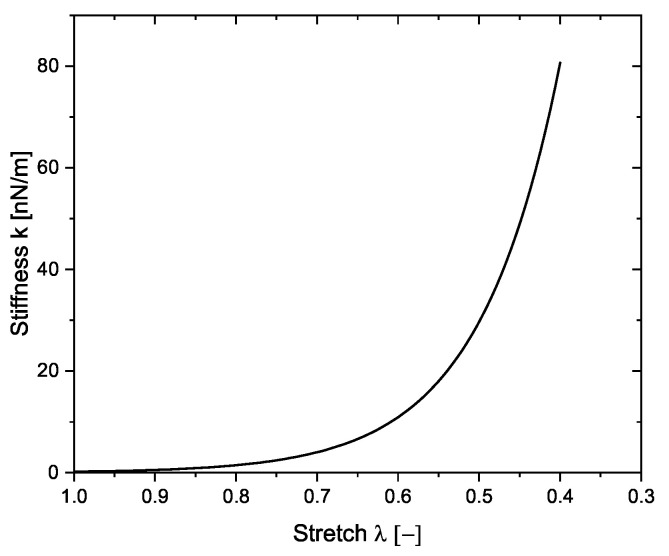
Evolution of the modeled stiffness in a collapsed cell with increasing compressive deformation.

In this work, a simple expression describing the stiffness *k* is given as

(8) $$k=k_{0}\exp\left(n\left(1-\lambda_{\zeta }\right)\right),$$

where $k_{0}$ is the initial stiffness in the spring. This equation is motivated by a molecular dynamics study on silica aerogels, where the average tangent modulus of the porous network was shown to evolve exponentially with respect to the applied strain [24]. The parameter *n* appears as a dimensionless-constant, and is considered to be equal to the absolute value of $d_{\mathrm{wall}}$ in nm. Thus, a thicker cell wall provides more resistance than a slenderer one. In the proposed model, affine deformation is used. Accordingly, $\lambda_{\zeta }=\lambda$, where λ is the micro-stretch in the network. Figure 2 shows the evolution of *k* with increasing compressive deformation λ.

### 2.2. Damage Evolution

**Figure 3:**
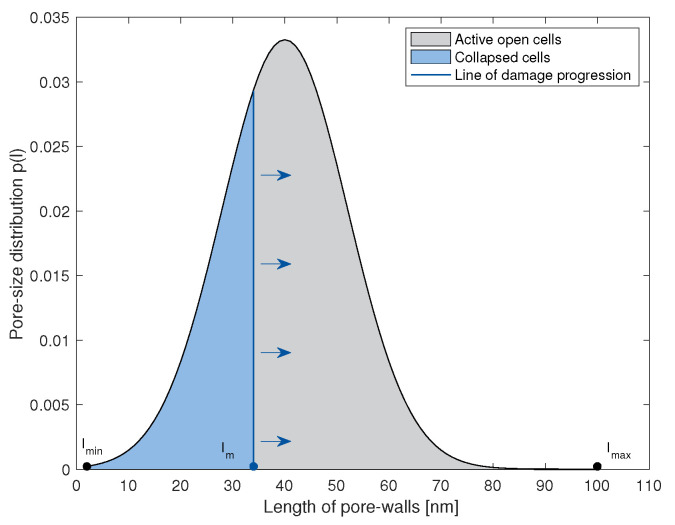
Illustration of the pore-size probability density function, showing the evolution of damage through the network and decomposition of the total network into active open and collapsed cells.

It was shown earlier [13], that the local bending and buckling of the cell walls is followed by a gradual collapse of the cells in the network. Beyond the instance of the first pore collapse, a reduction in the number of cells contributing to the elastic strain energy of the network is observed, as simultaneously, the number of collapsed cells begins to increase, starting with zero. Thus, the strain energy in the active network of cells and the collapsed network of cells can be expressed as

(9) $$\begin{array}{cc} \overset{d}{\Psi_{o}} & =N_{i}\int_{l_{m}\left(\overset{d}{\lambda }\right)}^{l_{max}}p\left(l\right)\psi_{o}\left(\overset{d}{\lambda },l\right)dl, \end{array}$$

(10) $$\begin{array}{cc} \overset{d}{\Psi_{c}} & =N_{i}\int_{l_{min}}^{l_{m}\left(\overset{d}{\lambda }\right)}p\left(l\right)\psi_{c}\left(\overset{d}{\lambda },l\right)dl, \end{array}$$

where p(l) and $N_{i}$ represent the cell-size distribution and the total number of initial cells in the network. Here, d represents an arbitrary spatial direction, and $\overset{d}{\lambda }$ denotes the micro-stretch in this direction, given by the identity $\overset{d}{\lambda }=\sqrt{dCd}$. There, C is the right Cauchy-Green tensor. Illustratively, the above-mentioned equations are graphically described in Figure 3. Thus, with increasing deformation $l_{m}\left(\overset{d}{\lambda }\right)$ moves towards $l_{max}$. In the reference configuration, $l_{m}\left(\overset{d}{\lambda }\right)=l_{min}$. This demonstrates the damage evolution through the network. As was shown in the previous work [8], for a given stretch value, the shorter cell walls fail sooner than the larger ones, when using the bending criterion. This is exhibited in Figure 3. p(l) can be any probability density function (PDF) that accurately describes the cell-size distribution of the material under consideration. In the past, normal [25] and generalized beta [11] PDFs have been used for modeling nanoporous cellular materials. In this study, for a sensitivity analysis of the parameters, a standard Gaussian distribution was assumed.

### 2.3. Directional Averaging

The above-formulated expressions describe a one-dimensional system. In order to generalize the strain energy to 3-d, the concept of directional averaging, as proposed by Bažant and Oh [26] has been used. It was shown, that such numerical integration schemes for directional averaging yield acceptable results in the case of open-porous materials [13,27]. To that end, the 3-d network strain energy can be described as

(11) $$\Psi =\frac{1}{A_{s}}\int_{S}\overset{d}{\Psi }d\overset{d}{y}\approx \sum_{m=1}^{n}\omega_{m}\overset{d_{m}}{\Psi },$$

where $\omega_{m}$ denotes the weight factors corresponding to the collocation directions $d_{m}$(m=1,2,3,…,n) over the half sphere. In this work, the numerical scheme proposed by Heo and Xu [28] with n=45 was used. Also, the 1-d total network strain energy $\overset{d}{\Psi }$ is the sum of the active $\overset{d}{\Psi_{o}}$ and the collapsed $\overset{d}{\Psi_{c}}$ networks. Finally, the first Piola-Kirchhoff stress tensor (T) is obtained as

(12) $$T=\frac{\partial \Psi }{\partial F}=\sum_{m=1}^{n}\omega_{m}\frac{\partial \overset{d_{m}}{\Psi }}{\partial \overset{d_{m}}{\lambda }}\frac{\partial \overset{d_{m}}{\lambda }}{\partial F},$$

where F is the deformation gradient. Affine deformation is assumed, such that the microscopic stretches follow the macroscopic deformation gradient.

## 3. Results and Discussion

**Figure 4:**
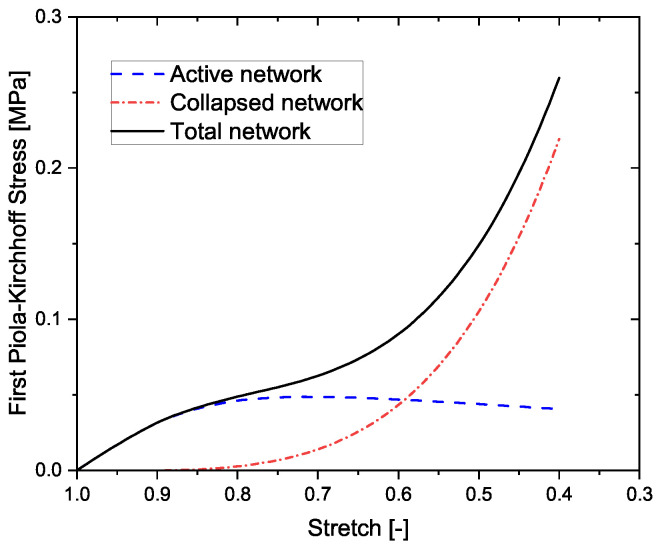
Breakdown of the networks and their effect on the macroscopic compressive stress-strain behaviour.

Figure 4 demonstrates the breakdown of the active and collapsed network responses together with the total network behavior. The active network governs the linear elastic regime and, upon damage, initiates the pore collapse. The slope of the linear elastic regime is dictated by the geometric and material characteristics of the struts, while the pore collapse is dictated by the damage mechanism, namely, elastic buckling, irreversible bending, and brittle collapse. At the first instance of pore collapse in the network, the collapsed network begins to play a role, which until then has no contribution to the total network strain energy. With increasing number of cells that collapse, the active network strongly softens, while the collapsed network correspondingly results in hardening. When neither of the network is dominating, a plateau appears in the stress-strain response. As soon as there are more collapsed cells in the network, compared to the active ones, the nature of the total network begins to harden. This can be termed as the onset of densification. The nature of hardening is then dependent on the stiffness of the spring. Generally, beyond a critical range of the number of cells, the total network response should be independent of the number of cells in the network. While this remains true, in our model, the parameters $N_{i}$, p(l) and $d_{\mathrm{wall}}$ together control the relative density of the material. The number of cells $N_{i}$ supports in describing the pore collapse and the densification in the network. Thus, it remains a multiplicative parameter in the model. To analyze the influence of different model parameters on the macroscopic behavior of the network, a parameter sensitivity analysis is presented in the following.

**Figure 5:**
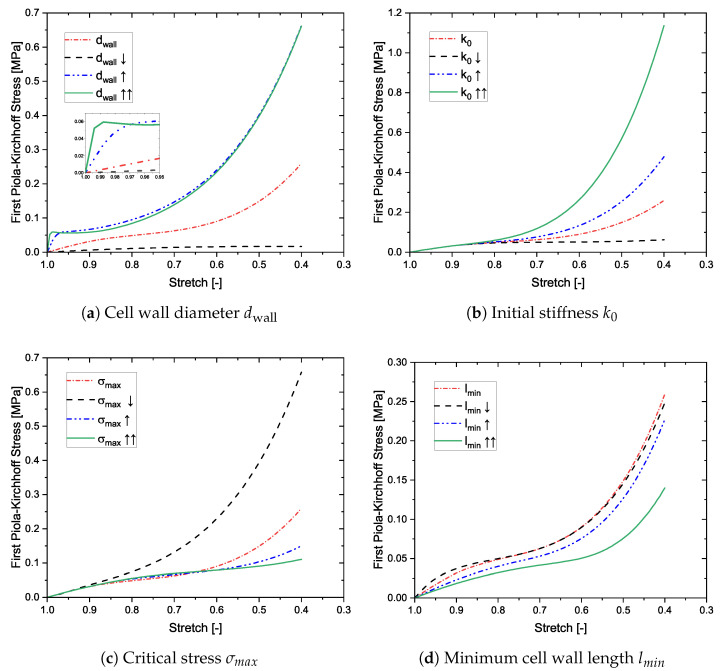
Sensitivity analysis of the model parameters towards the macroscopic stress-strain behavior.

Four parameters are chosen, namely, the diameter of the cell walls, the initial stiffness in the spring, the maximal allowable bending stress, and the minimum length of the struts. The diameter of the struts has a strong influence on the slope of the linear elastic regime (see Figure 5a). The model is capable of capturing diverse nature of the stress-strain curves. For e.g., the green solid curve captures the compressive behavior similar to that of a cancellous bone [29]. The pore collapse is also affected with changes to $d_{\mathrm{wall}}$ because any damage criteria, buckling or bending, is dependent on the slenderness of the cell wall. The collapsed network is not directly dependent on the cell wall diameter, but since the other two regimes are affected, so is the densification. Second, the stiffness $k_{0}$ is studied (see Figure 5b). This has no influence on the first two regimes, and thus using different values only affects the nature of hardening after pore collapse. Materials having different degrees of hardening can be modeled using this approach. Next, the influence of the maximal allowable bending stress is studied in Figure 5c. This parameter does not influence the linear elastic regime, but fully dictates the pore collapse. The initiation as well as the further progress of pore collapse is controlled by this parameter. Depending on the given value and the strut geometry, the pore collapse may be very slow, resulting in a longer plateau, or be quick, resulting in a sooner onset of densification. Another strut parameter *l* is of an interest to overall macroscopic behavior. Since in nanoporous materials, $d_{\mathrm{wall}}$ is generally closer to $l_{min}$ than $l_{max}$, the parameter $l_{min}$ is studied, and presented in Figure 5d. Higher the density of smaller cells, stiffer is the mechanical behavior. This echoes the results in literature [13]. Thus, the model is influenced by changes to the strut properties, both geometric and material, the mode of deformation, and the initial stiffness in the collapsed cells.

**Figure 6:**
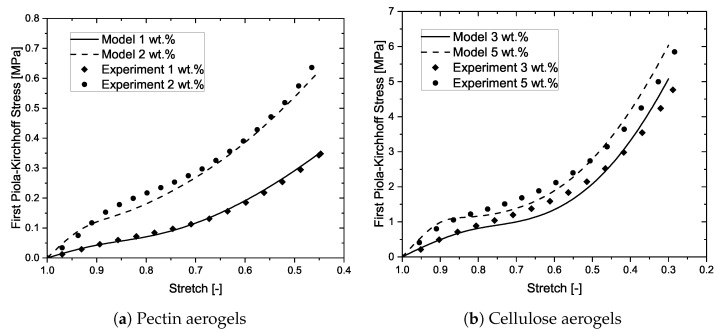
Model predictions vs. the experimental data of nanoporous materials.

After presenting the parameter sensitivity analysis, it is important to demonstrate whether the model is capable of describing the compressive stress-strain curve of some real porous materials, by comparing the model predictions with some experimental data. In this work, aerogels from pectin and cellulose were chosen. The experimental data is reused from a previous work [12], and the details on the synthesis and characterization methods together with the scanning electron microscopy images of the aerogels can be referred to in our previous works [8,12], for understanding the structure and appearance of the aerogel network. Two different concentrations of pectin aerogels and two different concentrations of cellulose aerogels have been modeled. Figure 6a,b exhibit the model predictions vs. the experimental data of the above-mentioned aerogels, respectively. The model shows very good agreement with the experimental data. All the three regimes are captured by the model. The model parameters remain the same as used in our previous model, except for the addition of the parameter $k_{0}$. These parameters are displayed in Table 1. The accuracy of the model is dependent on both, the physically-derived as well as the fitting, parameters. For example, the first four parameters in Table 1 are physically obtained from the experimental characterization of the aerogels, while the next two parameters are purely fitting ones. For the case of $N_{0}$, while the parameter is fitted to the experimental results, it is observed that with every increase in the wt.% concentration of the aerogels, the corresponding $N_{0}$ also increases. It was shown in Rege et al. [8], that increasing the wt.% concentration of polysaccharide-based aerogels results in increasing the density, thus increasing the number of cells. Thus, the changes in the parameter $N_{0}$ are qualitatively in agreement with the experimental observations. The discussion, as presented above, illustrates the capability of the model in describing real cellular materials. The effectiveness in the approach is attributed to the micromechanical nature of the model. Furthermore, upon close analysis, it is realized that the relation in Equation (2), may not necessarily hold for describing the onset of densification in nanoporous materials like aerogels. In the proposed model, the onset of densification can be considered to be the point where the stress-strain curves of the active and collapsed network intersect (see Figure 4). By further developing the proposed idea for modeling the collapsed cells, a more accurate description of the overall compressive behavior in all kinds of open-porous cellular materials can be obtained.

## 4. Conclusions

This paper presents a first-of-its-kind approach to model the densification behavior in open-porous cellular materials. The previously proposed generalized model for describing open-porous cellular materials [13] is extended to account for the hardening mechanism responsible for resulting in the compaction of the network. To this end, the collapsed cells are modeled as nonlinear springs. This now results in an additional component to the network strain energy. The material parameters remain the same as in the previous model with the only addition of the initial stiffness of the collapsed cell (spring). The model now presents a complete description of the compressive stress-strain response, namely, the linear elastic regime, the plateau regime and the densification one. The changes to the cell wall properties, both geometric and material, the mode of damage, and the initial stiffness in the collpased cells, are shown to dictate the overall mechanical behavior. The onset of densification is quantified by observing the point of intersection of the stress-strain curves corresponding to the active and collapsed networks. The model is validated against two different nanoporous aerogel-based materials, and it shows good agreement. 

## Figures and Tables

**Table 1:** Model parameters for pectin and cellulose aerogels corresponding to the data presented in Figure 6.

	$d_{\mathrm{wall}}$	$l_{\min}$	$l_{\max}$	$E_{\mathrm{skel}}$	$N_{0}$	$k_{0}$
	[nm]	[nm]	[nm]	[GPa]	[-]	[pN/m]
1 wt.% pectin	3.4	3.24	120.43	0.2	20 × $10^{21}$	7.0
2 wt.% pectin	3.5	3.26	105.76	0.2	60 × $10^{21}$	7.0
3 wt.% cellulose	5.5	3.01	64.25	12.0	22 × $10^{21}$	8.5
5 wt.% cellulose	6.4	3.37	109.40	12.0	23 × $10^{21}$	8.5

## Data Availability

Not applicable.
